# Lower heart rate in the early postoperative period does not correlate with long-term outcomes after repair of type A acute aortic dissection

**DOI:** 10.1007/s00380-014-0486-7

**Published:** 2014-02-25

**Authors:** Tetsu Ohnuma, Naoyuki Kimura, Yusuke Sasabuchi, Kayo Asaka, Junji Shiotsuka, Tetsuya Komuro, Hideyuki Mouri, Alan T. Lefor, Hideo Adachi, Masamitsu Sanui

**Affiliations:** 1 Department of Anesthesiology and Critical Care Medicine, Saitama Medical Center, Jichi Medical University, 1-847 Amanuma-cho, Omiya-ku, Saitama-shi, Saitama 330-8503 Japan; 2 Department of Cardiovascular Surgery, Saitama Medical Center, Jichi Medical University, Saitama, Japan; 3 Department of Surgery, Jichi Medical University, Shimotsuke, Japan

**Keywords:** Aortic dissection, Heart rate, Outcomes, Surgery

## Abstract

Little evidence exists regarding the need for a reduction in postoperative heart rate after repair of type A acute aortic dissection. This single-center retrospective study was conducted to determine if lower heart rate during the early postoperative phase is associated with improved long-term outcomes after surgery for patients with type A acute aortic dissection. We reviewed 434 patients who underwent aortic repair between 1990 and 2011. Based on the average heart rate on postoperative days 1, 3, 5, and 7, 434 patients were divided into four groups, less than 70, 70–79, 80–89, and greater than 90 beats per minute. The mean age was 63.3 ± 12.1 years. During a median follow-up of 52 months (range 16–102), 10-year survival in all groups was 67 %, and the 10-year aortic event-free rate was 79 %. The probability of survival and being aortic event-free using Kaplan–Meier estimates reveal that there is no significant difference when stratified by heart rate. Cox proportional regression analysis for 10-year mortality shows that significant predictors of mortality are age [Hazard Ratio (HR) 1.04; 95 % confidence interval (CI) 1.07–1.06; *p* = 0.001] and perioperative stroke (HR 2.30; 95 % CI 1.18–4.50; *p* = 0.024). Neither stratified heart rate around the time of surgery nor beta-blocker use at the time of discharge was significant. There is no association between stratified heart rate in the perioperative period with long-term outcomes after repair of type A acute aortic dissection. These findings need clarification with further clinical trials.

## Introduction

Acute aortic dissection (AAD) is a lethal cardiovascular disease. Despite continuing improvements in surgical and medical management, the 10-year survival rate in patients with type A AAD has remained low at 53.4–67.7 % [1–5]. Medical therapy to reduce blood pressure (BP) and heart rate in patients with AAD is widely used. Among medications, treatment with beta-blockers is reported as the key medical therapy by lowering both BP and cardiac contractility [6–8]. Heart rate is simply used as a surrogate of the degree of antiadrenergic and anti-inotropic effect of the drug therapy. Only one study has evaluated the efficacy of tight heart rate control in patients with type B AAD, which showed that the probability of aortic event-free survival was significantly higher in the tight heart rate control group compared with the conventionally managed heart rate group [8]. However, little evidence exists regarding the need for a reduction in postoperative heart rate after repair of type A AAD.

We conducted this study to evaluate whether a lower heart rate during the early postoperative phase improves the long-term outcomes after surgical repair of type A AAD.

## Materials and methods

### Study population

We retrospectively reviewed 434 patients who underwent surgical repair of a type A AAD at Jichi Medical University Saitama Medical Center between 1990 and 2011. Operations were performed urgently in all patients within 14 days of the onset of symptoms. The ethics committee approved this study, and the requirement for informed consent was waived. Patients who died within 6 days after surgery or had missing data, were excluded. Aortic dissection was proven by enhanced computed tomography, and defined as according to the Stanford and DeBakey classifications. We used data from the first operation during the hospitalization if more than one operation was performed. Preoperative variables evaluated in this study include age, gender, smoking, past medical history (hypertension, diabetes, dyslipidemia, chronic obstructive pulmonary disease, myocardial infarction, cardiac operation and bicuspid aortic valve), hypotension, aortic regurgitation, surgical parameters (duration of cardiopulmonary bypass, aortic cross clamp time, use of deep-hypothermic circulatory arrest), and laboratory data (hemoglobin and serum creatinine level). Postoperative events and outcomes include: the need for reoperation, aortic rupture in the hospital, atrial fibrillation, renal replacement therapy, stroke, prolonged ventilation (defined as the requirement ventilation for more than 48 h), tracheostomy, ICU length of stay, hospital length of stay and in-hospital mortality.

### Medical management and long-term follow-up

Intravenous beta-blockers and/or calcium channel blockers were administered to reduce systolic BP < 120 mmHg as initial therapy in the ICU after surgical repair. Intravenous medications were replaced by oral antihypertensive drugs including calcium channel blockers, beta-blockers, angiotensin-converting enzyme inhibitors, angiotensin receptor blockers and diuretics, as appropriate. The decision to give oral antihypertensive medications was at the discretion of the treating physician.

Heart rate and BP at 6 a.m., 2 p.m., and 8 p.m. daily during the hospitalization were recorded. Based on the average heart rate on postoperative days 1, 3, 5, and 7, 434 patients were divided into four groups, A: <70 beats per minute (bpm), B: 70–79 bpm, C: 80–89 bpm, and D: >90 bpm.

Follow-up data including survival time and cause of death once in every 3 years after repair of type A AAD were obtained by mailing a questionnaire to all patients. If responses were not returned, we contacted the patient, the patient’s family or the family physician by telephone. Aortic events were defined as aortic rupture, secondary operation, endovascular stenting, late organ ischemia or sudden death [8].

### Statistical analysis

Data are presented as mean ± standard deviation, medians (interquartile ranges, IQR) or percentages, as appropriate. Either Chi-square or Fisher’s exact test was used for nominal variables, and one-way analysis of variance was used to compare continuous variables. The Kruskal–Wallis test was used for nonparametric variables. A *p* value of <0.05 was considered statistically significant. Cox proportional hazard analysis was conducted to identify independent risk factors for 10-year mortality and aortic events. Cumulative 10-year survival rate and aortic event-free rate stratified by heart rate were analyzed with Kaplan–Meier analysis using the Log-rank test. All analysis was performed using SPSS 19.0 (IBM, USA).

## Results

**Fig. 1:**
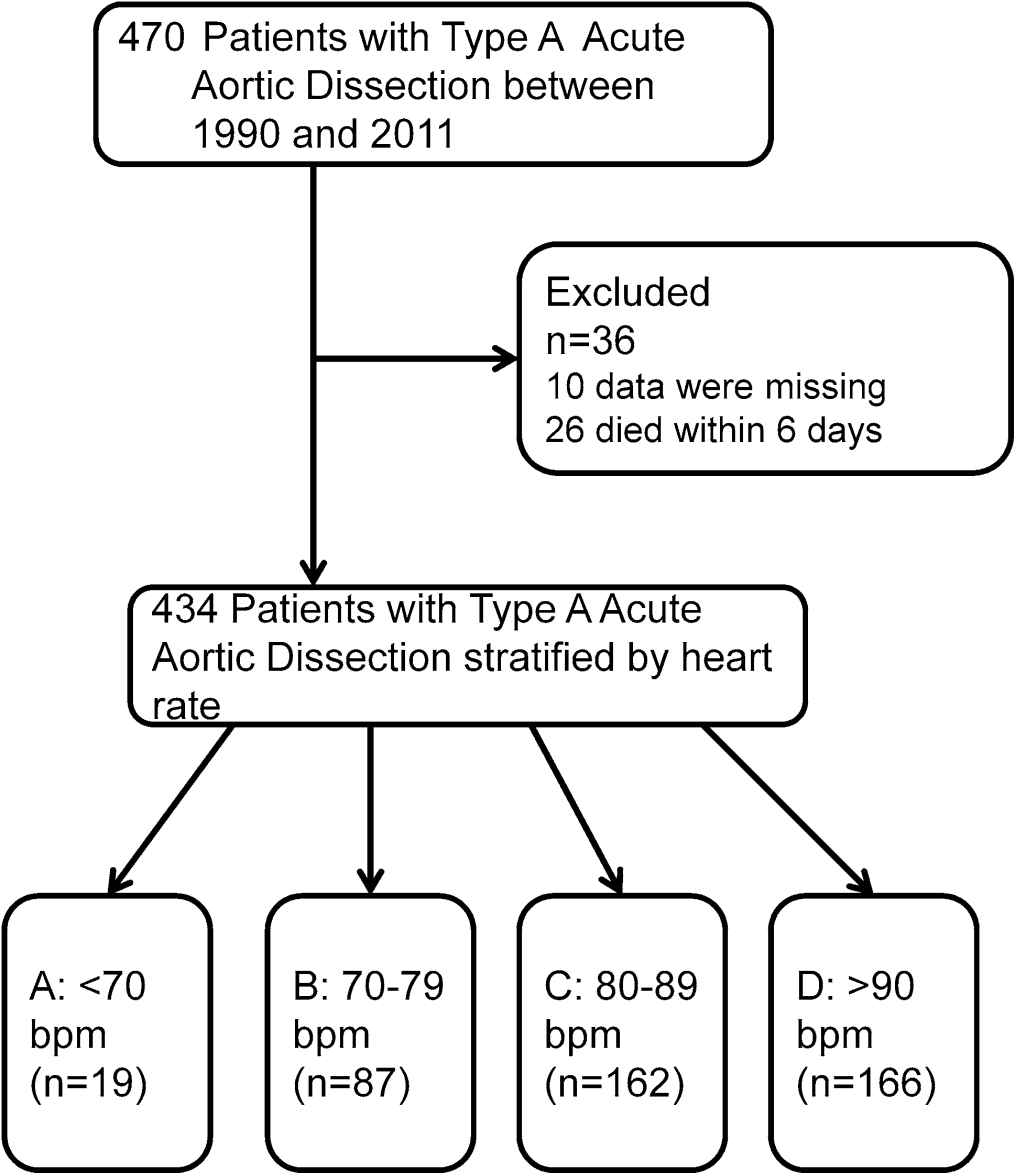
Patients included in the study, and the schema for classification into study Groups A, B, C and D, stratified by heart rate (bpm-beats per minute)

Four hundred and seventy patients underwent surgical repair of type A AAD. Twenty-six patients died within 6 days and data of 10 patients were missing, as shown in Fig. 1. Among the remaining 434 patients, the median follow-up period was 52 (IQR 16–102) months. The mean age was 63.3 ± 12.1 years, and the male: female ratio was 51:49. Preoperative comorbidities included hypertension (70 %), diabetes (6 %), dyslipidemia (18 %), chronic obstructive pulmonary disease (3 %), previous myocardial infarction (5 %), previous cardiac operation (1 %), Marfan syndrome (3 %), bicuspid aortic valve (1 %), hypotension (29 %), and aortic regurgitation (12 %).

Surgical procedures performed included hemi-arch replacement (23 %), aortic arch replacement (14 %), aortic valve replacement (1 %), aortic root replacement with Bentall procedure (4 %), coronary artery bypass grafting (7 %), and entry resection (73 %). The durations of operation, cardiopulmonary bypass time, aortic cross clamp time, and deep-hypothermic arrest time were 371 ± 128, 146 ± 52, 103 ± 39, and 35 ± 15 min, respectively. Postoperative complications included prolonged ventilation (>48 h) (40 %), atrial fibrillation (37 %), perioperative stroke (10 %), re-exploration for bleeding (6 %), need for renal replacement therapy (6 %), tracheostomy (4 %), and early postoperative aortic rupture (1 %).

The 434 patients were divided into four groups for further analysis based on their heart rate, including 19 patients (4 %) in Group A (heart rate <70 bpm), 87 patients (20 %) in Group B (70–79 bpm), 162 patients (37 %) in Group C (80–89 bpm), and 166 patients (38 %) in Group D (>90 bpm). No patient had an average heart rate less than 60 bpm. Postoperative average systolic and diastolic BP were 121 ± 10, 69 ± 7 mmHg, respectively. Tables 1 and 2 showed baseline and perioperative details categorized by the heart rate group. Significant differences between the four groups were seen in regard to dyslipidemia, preoperative serum creatinine, operative time, re-exploration for bleeding, atrial fibrillation, need for renal replacement therapy, prolonged ventilation, and postoperative average diastolic BP. ICU length of stay and hospital length of stay were also significantly different among the groups by univariate analysis.

**Table 1:** Baseline characteristics of patients with type A acute aortic dissection categorized by heart rate (*n* = 434)

Variable	Heart rate (bpm)				
	<70	70–79	80–89	>90	*p* value
Patient number	19	87	162	166	
Characteristics					
Male (%)	52.6	48.3	51.9	51.2	0.96
Age (years)	65 ± 10	64 ± 10	63 ± 13	63 ± 13	0.93
Smoking (%)	47.4	49.4	48.1	45.2	0.91
Medical history					
Hypertension (%)	89.5	75.9	67.9	66.9	0.11
Diabetes (%)	5.3	3.4	4.9	7.8	0.51
Dyslipidemia (%)	15.8	32.2	11.7	16.3	0.001
COPD (%)	0	1.1	2.5	4.8	0.41
Previous myocardial infarction (%)	10.5	4.6	3.7	4.8	0.61
Previous cardiac operation (%)	0	1.1	1.9	1.2	0.91
Marfan syndrome (%)	0	1.1	3.1	3.0	0.87
Bicuspid aortic valve (%)	0	2.3	0.6	0.6	0.45
Preoperative laboratory data					
Serum creatinine (mg/dl)	1.9 ± 2.8	1.2 ± 1.4	1.0 ± 0.6	1.0 ± 0.8	0.002
Hemoglobin (g/dl)	12.4 ± 2.0	12.1 ± 1.8	12.3 ± 2.0	12.3 ± 1.9	0.75
Preoperative shock (%)	15.8	26.4	33.3	27.7	0.36
Preoperative AR (%)	10.5	11.5	9.9	15.1	0.56
DeBakey classification					
Type I (%)	73.7	63.2	58.6	68.1	0.26
Type II (%)	5.3	11.5	16.7	7.2	0.05
Type IIIb retrograde (%)	21.1	23.0	24.1	24.7	0.98

*COPD* chronic obstructive pulmonary disease, *AR* aortic regurgitation

**Table 2:** Intraoperative and postoperative details and short-term outcomes of patients with type A acute aortic dissection categorized by heart rate

Variable	Heart rate (bpm)				
	<70	70–79	80–89	>90	*p* value
Surgery characteristics					
Operation time (min)	351 ± 125	337 ± 96	363 ± 114	398 ± 150	0.003
CPB time (min)	148 ± 72	142 ± 45	142 ± 48	151 ± 59	0.34
Aorta cross clamp time (min)	106 ± 50	105 ± 35	101 ± 34	103 ± 44	0.89
DHCA time (min)	35 ± 20	35 ± 14	35 ± 14	34 ± 16	0.99
Hemi-arch replacement (%)	26.3	31.0	22.8	17.5	0.10
Aortic arch replacement (%)	21.1	13.8	9.9	16.9	0.20
Aortic valve replacement (%)	5.3	0	1.2	1.2	0.29
Bentall (%)	0	3.4	4.9	3.6	0.89
CABG (%)	0	6.9	4.9	9.6	0.29
Entry resection (%)	78.9	75.9	72.8	71.5	0.83
Re-exploration for bleeding (%)	0	1.1	4.3	9.6	0.024
Postoperative events					
Aortic rupture in hospital (%)	0	1.1	0.6	0.6	1.00
Atrial fibrillation (%)	15.8	29.9	34.0	46.7	0.005
Renal replacement therapy (%)	10.5	3.4	3.1	9.6	0.037
Perioperative stroke (%)	10.5	8.0	8.6	13.3	0.49
Prolonged ventilation (%)	47.4	32.6	33.8	50.0	0.008
Tracheostomy (%)	0	4.6	3.7	3.0	0.85
Postoperative average SBP (mmHg)	118 ± 9	121 ± 10	121 ± 11	121 ± 10	0.54
Postoperative average DBP (mmHg)	65 ± 8	67 ± 6	69 ± 7	70 ± 7	<0.001
Short-term outcomes					
ICU stay (days)	5 (3–10)	5 (4–7)	5 (4–7)	7 (5–10)	<0.001
Hospital stay (days)	20 (15–31)	20 (16–28)	23 (18–30)	25 (19–35)	0.005
Hospital mortality (%)	0	2.3	5.6	4.2	0.63

*CPB* cardiopulmonary bypass, *DHCA* deep-hypothermic circulatory arrest, *CABG* coronary artery bypass graft, *SBP* systolic blood pressure, *DBP* diastolic blood pressure

The postoperative medications are shown in Table 3. Most patients in the study (91 %) required at least one antihypertensive medication at the time of discharge, which included beta-blockers (54 %), angiotensin-converting enzyme inhibitors (11 %), angiotensin receptor blockers (32 %), calcium channel blockers (62 %), and diuretics (28 %). Beta-blocker use was significantly associated with a lower heart rate (*p* < 0.001).

**Table 3:** Postoperative medications

	Heart rate (bpm)					
Variable	Total	<70	70–79	80–89	>90	*p* Value
Beta-blockers						
Initiation within 7 days after operation (%)	52.1	73.7	70.6	58.2	32.7	<0.001
At discharge (%)	53.8	68.4	68.2	59.5	39.0	<0.001
ACE inhibitors at discharge (%)	11.3	15.8	8.2	9.2	14.5	0.31
ARB at discharge (%)	32.2	52.6	44.7	28.1	27.0	0.006
CCBs at discharge	62.5	78.9	68.2	58.8	61.0	0.23
Diuretics at discharge	27.6	26.3	14.1	27.5	35.2	0.004

*ACE* angiotensin-converting enzyme, *ARB* angiotensin receptor blocker, *CCB* calcium channel blocker

**Fig. 2:**
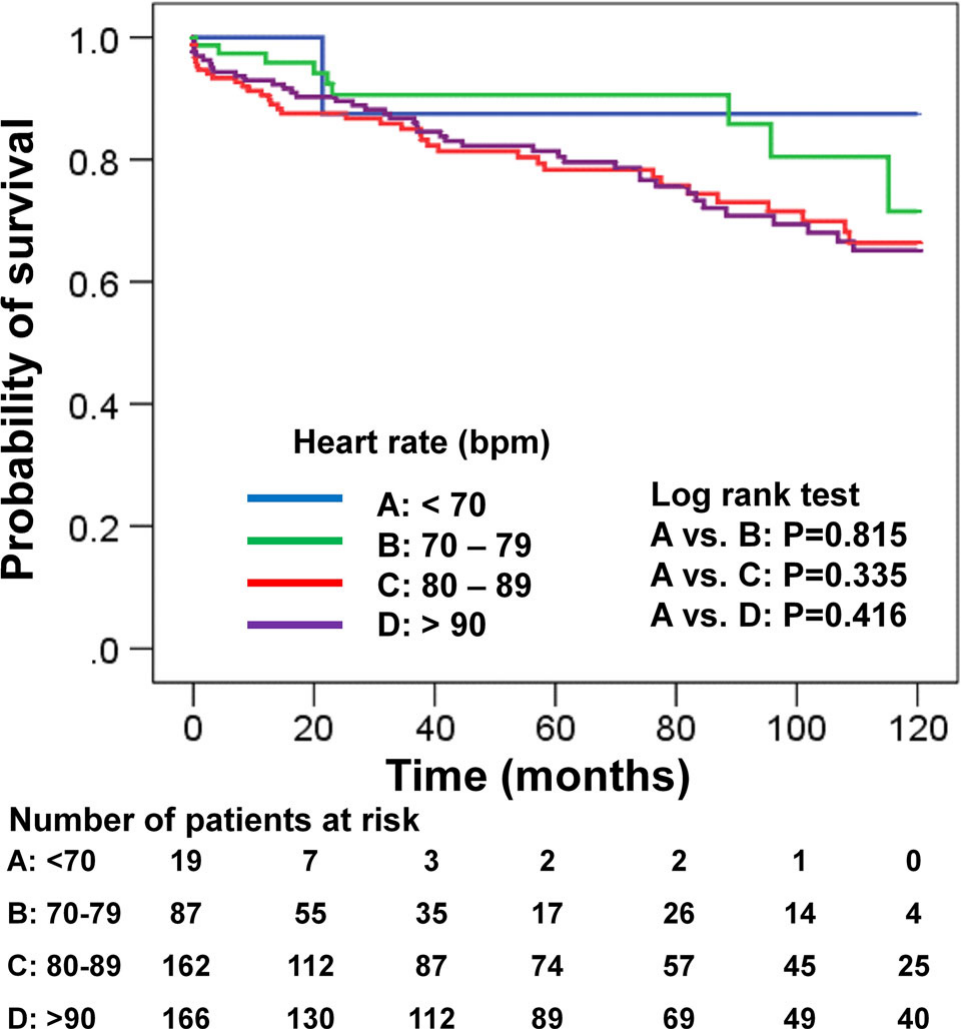
Kaplan–Meier curves for overall 10-year survival in patients with type A acute aortic dissection, stratified by heart rate

**Fig. 3:**
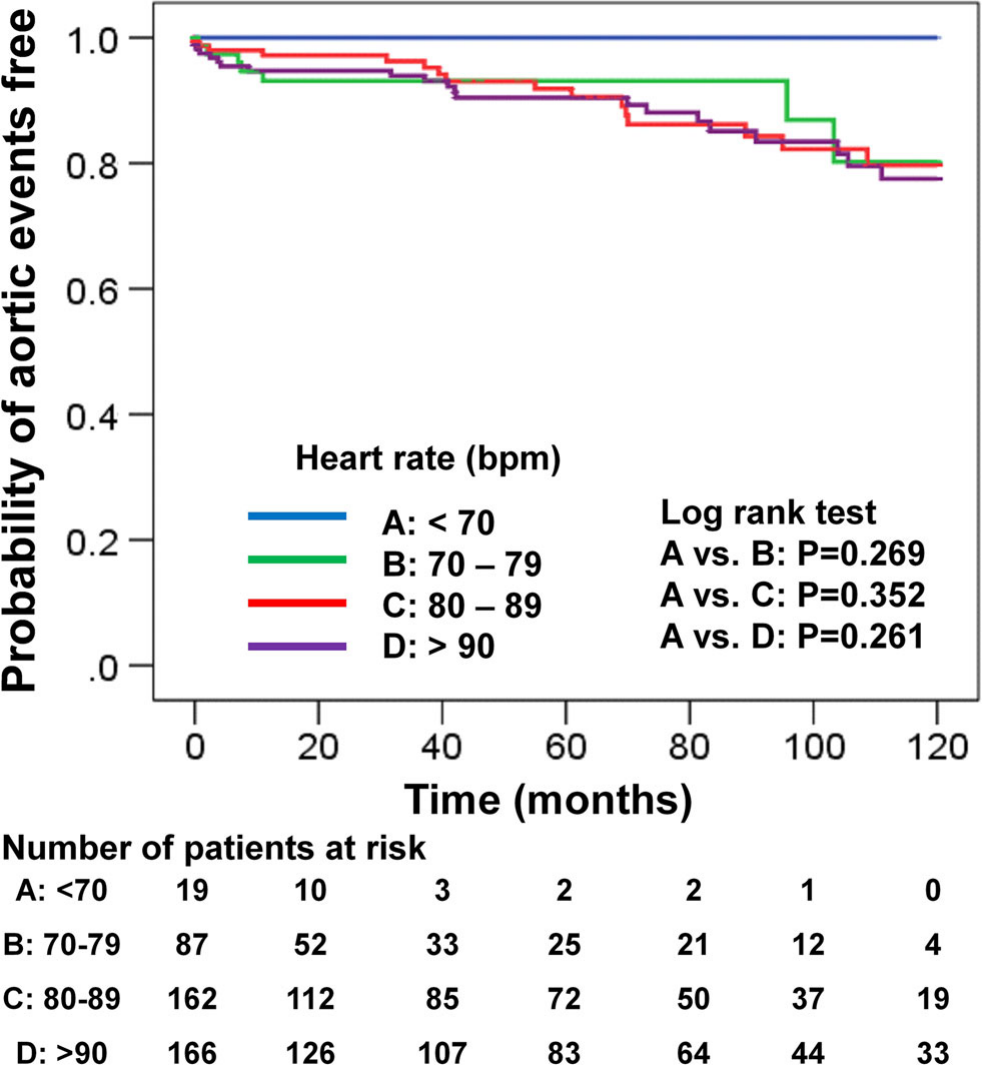
Kaplan–Meier curves for 10-year aortic event-free survival in patients with type A acute aortic dissection, stratified by heart rate

Cumulative survival rate from the time of admission in all groups was 97 % at 1 month, 93 % at 1 year, 81 % at 5 years, and 67 % at 10 years. Cumulative aortic event-free rate in all groups was 98 % at 1 month, 96 % at 1 year, 91 % at 5 years, and 79 % at 10 years. The probability of 10-year survival categorized by heart rate using Kaplan–Meier analysis reveals no significant differences among the groups (A vs. B, *p* = 0.815; A vs. C, *p* = 0.335; A vs. D, *p* = 0.415), as shown in Fig. 2. The probability of being 10-year aortic event-free was also not significantly different among the groups (A vs. B, *p* = 0.269; A vs. C, *p* = 0.352; A vs. D, *p* = 0.261), as shown in Fig. 3. Cox proportional regression analysis for 10-year mortality showed that significant predictors of mortality include age [Hazard Ratio (HR) 1.04; 95 % confidence interval (CI) 1.07–1.06; *p* = 0.001] and perioperative stroke (HR 2.30; 95 % CI 1.18–4.50; *p* = 0.024) (Table 4). Neither stratified heart rate nor beta-blocker use was significant. Cox proportional regression analysis for 10-year aortic events was also performed. Only a diagnosis of Marfan syndrome was a significant risk factor in that model (HR 6.59; 95 % CI 2.57–16.87; *p* < 0.001).

**Table 4:** Multivariate analysis of risk factors for 10-year mortality and aortic events

	*p* value	Hazard ratio	95 % CI
Variable			
10-year mortality			
Age	0.001	1.038	1.016–1.061
Perioperative stroke	0.015	2.302	1.177–4.503
10-year aortic events			
Marfan syndrome	<0.001	6.591	2.574–16.872

*CI* confidence interval

## Discussion

In this study, we examined the association of heart rate, stratified into four groups, in the early postoperative period with long-term outcomes after repair of type A AAD. The key findings showed that there is no significant difference in 10-year survival among the four groups using Kaplan–Meier analysis, nor is there a difference in the probability of being 10-year aortic event-free. Cox regression analysis showed that significant predictors of 10-year mortality include age and perioperative stroke. Only a diagnosis of Marfan syndrome was significant in the model for 10-year aortic events. Neither stratified heart rate nor any medication was a significant predictor for both 10-year mortality and aortic events.

Patients with Type A AAD usually undergo surgical repair, and postoperative medical therapy is routinely given to stabilize the aortic wall and prevent aortic rupture and recurrent dissection [9]. Medical management of AAD was proposed in classic studies in the 1960s [10, 11]. In those studies, authors established two primary goals of medical therapy including reduction in BP and diminution of left ventricular ejection force (dp/dt). Among medications, beta-blockers have been a key drug for the control of both dp/dt and BP by reducing both the heart rate and BP [12]. Reasonable targets for heart rate and systolic BP have been less than 60 bpm and between 100 and 120 mm Hg, respectively [12]. Until the present, however, use of these medical therapies for AAD has not been supported by solid clinical evidence.

There is only one study that investigated the effect of tight heart rate control in patients with type B AAD [8]. Kodama and colleagues retrospectively collected 171 patients with type B AAD treated medically in a single center between 1997 and 2005. Based on the average heart rate at 3, 5, and 7 days after the onset, patients were divided into a tight heart rate management group (<60 bpm, *n* = 32) and a control group (≥60 bpm, *n* = 139). The main outcome of Kaplan–Meier analysis for aortic event-free survival (median follow-up 27 months) showed that the tight heart rate control group had a significantly higher rate of being aortic event-free than the conventional heart rate control group (*p* = 0.017). In comparison, the current study did not show a significant difference in long-term outcomes when stratified by heart rate. While this discrepancy may be caused by physiologic differences between type A AAD and type B AAD, studies to date have not conclusively shown whether or not we need to further reduce the heart rate.

In patients who underwent repair of type A AAD, little was known about the effects of medical therapy on long-term survival [13, 14]. In contrast, there have been several studies to evaluate the long-term outcomes of medical therapy in patients with type B AAD [7, 8, 13, 15–17]. Suzuki and colleagues [13] investigated the effects of medications on long-term outcomes in 1301 patients (722 with type A and 579 with type B) using the International Registry of Acute Aortic Dissection (IRAD) database from 1995 with follow-up to 5 years. The results of multivariate analysis of 654 surgically treated patients with type A AAD showed that beta-blocker use was significantly associated with lower mortality (HR 0.47, 95 % CI 0.25–0.90, *p* = 0.02). Calcium channel blockers and renin–angiotensin system inhibitors were not included in their model. There have been reports that calcium channel blockers’ use is significantly associated with improved outcomes for patients with type B AAD [13, 16]. Regarding the present study, however, no other studies have shown a positive effect of long-term calcium channel blockers’ use on patients with type A AAD. Although renin–angiotensin system inhibitors might be useful in patients with Marfan syndrome and aortic aneurysms [18, 19], no evidence exists regarding the effects of these medications on patients with Type A AAD.

As previously mentioned, BP control is the other primary goal of medical therapy in patients with type A AAD. A recent single-center study evaluated the impact of BP on 25-year outcomes following repair of type A AAD between 1984 and 2009 [3]. Based on the BP at late follow-up, all 252 patients were divided into three groups: systolic BP < 120, 120–140, and >140 mmHg. Patients who maintained a systolic BP < 120 mmHg had improved freedom from reoperation compared with those having a BP 120–140 or >140 mmHg (*p* < 0.001) by Kaplan–Meier analysis. The present study failed to show an association between postoperative systolic BP in the acute phase and, either 10-year mortality, or being aortic event-free, by Cox regression analysis. Although, we have not been able to investigate the effect of the late-phase BP in the type A AAD patients reviewed here, late follow-up of BP might be an important factor in addition to postoperative BP.

Marfan syndrome is one of the major inherited connective tissue disorders that may lead to the weakening of the aortic media, and subsequently to AAD, aortic aneurysm or rupture. Previous clinical trials showed that Marfan syndrome was one of the risk factors for late aortic events [20, 21]. In the current study, Marfan syndrome was included among the risk factors for the occurrence of long-term aortic events. This might be partially explained by the fact that the dissected aortic wall in patients with Marfan syndrome tends to develop aneurysmal dilatation due to wall weakness, more than in type A AAD patients [22].

There are several acknowledged limitations to this study. Most importantly, we evaluated only the acute phase of the average heart rate and BP on postoperative days 1, 3, 5, and 7, and we did not investigate the heart rate, BP or medications during long-term follow-up. Approximately, half of the patients after discharge are followed at regular hospital visits, and the remaining patients are followed in other hospitals or clinics. Thus, data for only about half of the patients regarding heart rate, BP, and medications are available after discharge from the hospital. The protective factor of heart rate and BP is likely cumulative over time. The time points during the acute postoperative period may not be adequate surrogates for long-term control of heart rate, because the long-term heart rate and BP are probably different compared to those in the immediate postoperative period. These parameters may contribute to the failure to show a long-term difference in survival. Secondly, the present study is a single-center retrospective study. Although the sample size is not small compared with previous studies reporting long-term outcomes in patients with type A AAD [1–5, 20, 23–25], these findings may be limited in application to other type A AAD patients because of the relatively homogenous population. Thirdly, the control of heart rate was not sufficient in our population because no one achieved less than 60 bpm and only 19 patients were less than 70. Although patients with beta-blockers tended to have a lower heart rate as shown in Table 3, it is difficult to reduce the heart rate after surgical repair due to multiple factors including, inflammation, infection, and atrial fibrillation. Fourthly, class effects and dosages of the each medication were not reviewed in this study. We did not test the effect of the combination of drugs that might have more synergy. Furthermore, there was a lack of information regarding additional medications and compliance. The medications at discharge may have been changed later in the study period. It is acknowledged that the medication list at the time of discharge may not adequately reflect long-term medication usage in the study population. Finally, the choice of antihypertensive drugs was made by the individual treating physicians. Selection bias potentially occurred even after adjusting for the risk factors.

## Conclusions

We did not observe a difference in the long-term mortality or incidence of aortic events in patients after repair of type A AAD, when stratified by heart rate during the acute hospitalization. There are several possible explanations for this observation. It remains unknown whether lower heart rate is important for outcomes. These findings suggest the need for further study in randomized clinical trials.

## References

[1] Bekkers JA, Bol Raap G, Takkenberg JJ, Bogers AJ (2013). Acute type A aortic dissection: long-term results and reoperations. Eur J Cardiothorac Surg.

[2] Gariboldi V, Grisoli D, Kerbaul F, Giorgi R, Riberi A, Metras D, Mesana TG, Collart F (2007). Long-term outcomes after repaired acute type A aortic dissections. Interact CardioVasc Thorac Surg.

[3] Melby SJ, Zierer A, Damiano RJ, Moon MR (2013). Importance of blood pressure control after repair of acute type a aortic dissection: 25-year follow-up in 252 patients. J Clin Hypertens (Greenwich).

[4] Stevens LM, Madsen JC, Isselbacher EM, Khairy P, MacGillivray TE, Hilgenberg AD, Agnihotri AK (2009) Surgical management and long-term outcomes for acute ascending aortic dissection. J Thorac Cardiovasc Surg 138(6):1349–1357 e1341 10.1016/j.jtcvs.2009.01.030 19660400

[5] Tan ME, Morshuis WJ, Dossche KM, Kelder JC, Waanders FG, Schepens MA (2005). Long-term results after 27 years of surgical treatment of acute type a aortic dissection. Ann Thorac Surg.

[6] Shores J, Berger KR, Murphy EA, Pyeritz RE (1994). Progression of aortic dilatation and the benefit of long-term beta-adrenergic blockade in Marfan’s syndrome. N Engl J Med.

[7] Genoni M, Paul M, Jenni R, Graves K, Seifert B, Turina M (2001). Chronic beta-blocker therapy improves outcome and reduces treatment costs in chronic type B aortic dissection. Eur J Cardiothorac Surg.

[8] Kodama K, Nishigami K, Sakamoto T, Sawamura T, Hirayama T, Misumi H, Nakao K (2008). Tight heart rate control reduces secondary adverse events in patients with type B acute aortic dissection. Circulation.

[9] Taguchi E, Nishigami K, Miyamoto S, Sakamoto T, Nakao K (2013). Impact of shear stress and atherosclerosis on entrance-tear formation in patients with acute aortic syndromes. Heart Vessels.

[10] Wheat MW, Palmer RF, Bartley TD, Seelman RC (1965). Treatment of dissecting aneurysms of the aorta without surgery. J Thorac Cardiovasc Surg.

[11] Austen WG, DeSanctis RW (1966). Dissecting aneurysm. Surg Clin North Am.

[12] Tsai TT, Nienaber CA, Eagle KA (2005). Acute aortic syndromes. Circulation.

[13] Suzuki T, Isselbacher EM, Nienaber CA, Pyeritz RE, Eagle KA, Tsai TT, Cooper JV, Januzzi JL, Braverman AC, Montgomery DG, Fattori R, Pape L, Harris KM, Booher A, Oh JK, Peterson M, Ramanath VS, Froehlich JB (2012). Type-selective benefits of medications in treatment of acute aortic dissection (from the International Registry of Acute Aortic Dissection [IRAD]). Am J Cardiol.

[14] Zierer A, Voeller RK, Hill KE, Kouchoukos NT, Damiano RJ, Moon MR (2007). Aortic enlargement and late reoperation after repair of acute type A aortic dissection. Ann Thorac Surg.

[15] Estrera AL, Miller CC, Safi HJ, Goodrick JS, Keyhani A, Porat EE, Achouh PE, Meada R, Azizzadeh A, Dhareshwar J, Allaham A (2006). Outcomes of medical management of acute type B aortic dissection. Circulation.

[16] Sakakura K, Kubo N, Ako J, Fujiwara N, Funayama H, Ikeda N, Nakamura T, Sugawara Y, Yasu T, Kawakami M, Momomura S (2009). Determinants of long-term mortality in patients with type B acute aortic dissection. Am J Hypertens.

[17] Delsart P, Beregi JP, Devos P, Haulon S, Midulla M, Mounier-Vehier C (2013). Thrombocytopenia: an early marker of late mortality in type B aortic dissection. Heart Vessels.

[18] Brooke BS, Habashi JP, Judge DP, Patel N, Loeys B, Dietz HC (2008). Angiotensin II blockade and aortic-root dilation in Marfan’s syndrome. N Engl J Med.

[19] Mochizuki S, Dahlof B, Shimizu M, Ikewaki K, Yoshikawa M, Taniguchi I, Ohta M, Yamada T, Ogawa K, Kanae K, Kawai M, Seki S, Okazaki F, Taniguchi M, Yoshida S, Tajima N (2007). Valsartan in a Japanese population with hypertension and other cardiovascular disease (Jikei Heart Study): a randomised, open-label, blinded endpoint morbidity-mortality study. Lancet.

[20] Fattouch K, Sampognaro R, Navarra E, Caruso M, Pisano C, Coppola G, Speziale G, Ruvolo G (2009). Long-term results after repair of type a acute aortic dissection according to false lumen patency. Ann Thorac Surg.

[21] Yu HY, Chen YS, Huang SC, Wang SS, Lin FY (2004). Late outcome of patients with aortic dissection: study of a national database. Eur J Cardiothorac Surg.

[22] Dean JC (2002). Management of Marfan syndrome. Heart.

[23] Evangelista A, Salas A, Ribera A, Ferreira-Gonzalez I, Cuellar H, Pineda V, Gonzalez-Alujas T, Bijnens B, Permanyer-Miralda G, Garcia-Dorado D (2012). Long-term outcome of aortic dissection with patent false lumen: predictive role of entry tear size and location. Circulation.

[24] Modi A, Vohra HA, Kaarne M, Haw MP, Barlow CW, Ohri SK, Livesey SA, Tsang GM (2011). Long-term outcome following repair of acute type A aortic dissection after previous cardiac surgery. Interact CardioVasc Thorac Surg.

[25] Tsai TT, Evangelista A, Nienaber CA, Trimarchi S, Sechtem U, Fattori R, Myrmel T, Pape L, Cooper JV, Smith DE, Fang J, Isselbacher E, Eagle KA (2006). Long-term survival in patients presenting with type A acute aortic dissection: insights from the International Registry of Acute Aortic Dissection (IRAD). Circulation.

